# Adiponectin and All-Cause Mortality in Patients with Chronic Kidney Disease: A Systematic Review and Meta-Analysis

**DOI:** 10.3390/metabo15040230

**Published:** 2025-03-27

**Authors:** Hyun Suk Yang, Soo-Nyung Kim, Jung-Hoon Ro, Mina Hur

**Affiliations:** 1 Department of Cardiovascular Medicine, Research Institute of Medical Science, Konkuk University School of Medicine, Seoul 05029, Republic of Korea; yang.hyun@kuh.ac.kr; 2 Department of Obstetrics and Gynecology, Konkuk University School of Medicine, Seoul 05029, Republic of Korea; dr.snkim@gmail.com; 3 Department of Biomedical Engineering, Pusan National University School of Medicine, Pusan National University Hospital, Busan 49241, Republic of Korea; jhro@pnu.edu; 4 Department of Laboratory Medicine, Konkuk University School of Medicine, Konkuk University Medical Center, Seoul 05030, Republic of Korea

**Keywords:** adiponectin, chronic kidney disease, hemodialysis, peritoneal dialysis, all-cause mortality

## Abstract

**Background/Objectives**: Elevated levels of adiponectin in chronic kidney disease (CKD) have been paradoxically associated with increased mortality. This meta-analysis aimed to evaluate the association between circulating adiponectin levels and all-cause mortality in patients with CKD, in total and various subgroups. **Methods**: We systematically searched PubMed, Embase, and Cochrane Library from their inception to December 2024 for studies examining baseline adiponectin levels and observed mortality outcomes in patients with CKD. Studies were included if they evaluated CKD stages 2–5 patients, measured baseline circulating adiponectin levels, and reported hazard ratios (HRs) for all-cause mortality. We excluded non-original research, studies of acute conditions, normal kidney function, kidney transplantation, and those using log-transformed or standardized HRs. HRs with a 95% confidence interval (CI) for all-cause mortality risk per 1 µg/mL increase in adiponectin were extracted and analyzed using the Comprehensive Meta-Analysis Version 4. Study quality was assessed using the Newcastle–Ottawa Scale. **Results**: Twelve studies with 2523 subjects were included. The pooled unadjusted HR was 1.003 (95% CI: 0.981–1.025) using a random-effects model (*I*^2^ = 79%). Subgroup analyses demonstrated increased mortality risk with elevated adiponectin levels in non-Asia (HR 1.021 [95% CI: 1.006–1.037], *p* = 0.006), studies with female proportion <47% (HR 1.021 [95% CI: 1.009–1.033], *p* < 0.001), and studies with body mass index ≥25 kg/m^2^ (HR 1.023 [95% CI: 1.008–1.038], *p* = 0.003). In contrast, higher adiponectin levels were associated with decreased mortality risk in the peritoneal dialysis group (HR 0.956 [95% CI: 0.934–0.979], *p* < 0.001) and female proportion ≥47% group (HR 0.929 [95% CI: 0.874–0.988], *p* = 0.019). **Discussion/Conclusions**: This meta-analysis revealed that elevated adiponectin levels have varying associations with the risk of all-cause mortality across CKD patient subgroups. These findings suggest that the prognostic value of adiponectin levels in CKD may be modulated by demographic and clinical factors. Limitations include poor generalizability with underrepresentation of early-stage CKD. This research received no external funding and was not registered.

## 1. Introduction

Adiponectin, an adipokine primarily secreted by adipose tissue, plays crucial roles in metabolic signaling and cellular communication [1,2]. It acts through ceramidase-active AdipoR1/R2 receptors, T-cadherin-mediated exosome release, and cross-tissue signaling that collectively enhance insulin sensitivity and reduce inflammation [3,4]. Adiponectin has garnered attention for its beneficial effects, including improved glucose metabolism and protection against metabolic and cardiovascular diseases [5,6,7,8].

In the context of chronic kidney disease (CKD), adiponectin levels present a clinical paradox. While plasma adiponectin typically ranges from 2 to 20 µg/mL in healthy individuals, patients with CKD exhibit levels 2–3 times higher than normal. This elevation is attributed primarily to decreased renal clearance, though metabolic disturbances and adiponectin resistance may also contribute [9]. The clinical significance of elevated adiponectin in CKD patients remains unclear as to whether it merely reflects impaired kidney function or serves as a prognostic indicator for clinical outcomes [10,11].

Despite adiponectin’s generally protective effects, several studies have documented an “adiponectin paradox”, where higher levels correlate with increased cardiovascular and all-cause mortality [12,13]. In CKD patients, specifically, the relationship between adiponectin and mortality risk remains controversial, with studies reporting positive [14,15], negative [16], or no association [17] with all-cause mortality. While no direct causal mechanism has been established between adiponectin and mortality, potential pathways linking elevated adiponectin to adverse outcomes in CKD patients include protein-energy wasting syndrome, detrimental cardiovascular effects, and altered immunometabolism in the uremic milieu [18,19].

A previous meta-analysis examining mortality risk factors in hemodialysis (HD) patients reported that adiponectin (per 10.0 µg/mL increment) was associated with increased all-cause mortality risk (relative risk: 1.23, 95% confidence interval [CI]: 1.08–1.41, *p* = 0.002) [20]. However, this analysis was limited by the small number of studies specifically investigating adiponectin as a biomarker [21].

Given these conflicting findings and the limited scope of previous analyses, we conducted a comprehensive systematic review and meta-analysis to evaluate the association between circulating adiponectin levels and all-cause mortality in CKD patients, examining both overall effects and various subgroup analyses

## 2. Materials and Methods

This systematic review and meta-analysis were conducted in accordance with the Preferred Reporting Items for Systematic Reviews and Meta-Analyses (PRISMA) guidelines [22] (Supplementary Materials Tables S1 and S2), the Cochrane Handbook for Systematic Reviews [23], and Meta-analysis of Observational Studies in Epidemiology (MOOSE) reporting guidelines [24].

### 2.1. Literature Search

We conducted a systematic electronic search of multiple databases including PubMed, Embase, and the Cochrane Library from their inception to December 2024. The search was restricted to English-language publications. We used the following search terms: (adiponectin) and (“chronic kidney/renal disease” or “chronic kidney/renal failure” or “chronic renal insufficiency” or “end stage kidney/renal disease” or “hemodialysis” or “peritoneal dialysis”) and (“death” or “mortality”). References from relevant articles were also reviewed.

### 2.2. Study Selection

Original articles were included if they met all of the following criteria: (1) evaluated patients with CKD stages 2–5 as defined by the Kidney Disease Improving Global Outcomes (KDIGO) guidelines [25], (2) measured baseline circulating total adiponectin levels, and (3) reported a hazard ratio (HR) with a 95% CI for all-cause mortality risk associated with adiponectin levels.

Exclusion criteria were: (1) non-original research formats (reviews, editorials, letters, conference abstracts, or case reports), (2) studies of subjects with acute medical conditions, normal glomerular filtration rate, or history of kidney transplantation, and (3) studies reporting an HR using log-transformed values or standard deviation units. For multiple publications using the same cohort, we included only the report with the largest sample size. Two independent reviewers (HSY and JHR) performed the study selection process. Any disagreements were resolved through consensus discussion or consultation with a third reviewer (SNK).

### 2.3. Data Extraction and Quality Assessment

From each eligible study, we extracted: (1) first author’s last name, (2) publication year, (3) study site, (4) CKD stage or dialysis modality, (5) sample size (total and by sex), (6) mean age, (7) baseline body mass index (BMI), (8) adiponectin measurement methodology, (9) baseline total adiponectin levels, (10) follow-up duration, (11) mortality count, (12) unadjusted and adjusted HR (95% CI) for all-cause mortality, and (13) adjustment variables. No attempts were made to contact study authors for additional information, and no automation tools were used in the data extraction. Study quality was assessed using the Newcastle–Ottawa Scale (NOS) for Cohort Studies [26], which is specifically designed and widely validated for observational research. Two reviewers (HSY, JHR) independently collected data from reports and evaluated each NOS criterion, with any disagreements resolved through discussion and consultation with a third reviewer (SNK).

### 2.4. Statistical Analysis

For the meta-analysis, we collected unadjusted HRs and their 95% CI for all-cause mortality risk per 1 µg/mL increase in adiponectin levels. For data from Rhee et al. [17], we standardized the HR from per 10 µg/mL to per 1 µg/mL using the formula: HR (per 1-unit) = exp (ln (HR per 10-units)/10) [27]. Data from Takemoto et al. [28], were stratified by sex and analyzed separately as ‘Takemoto 2009a’ (males) and ‘Takemoto 2009b’ (females), as the original study reported sex-specific analyses without combined data.

We conducted the meta-analysis using Comprehensive Meta-Analysis software (version 4.0) [29]. The pooled mean effect size was calculated using a random-effects model based on unadjusted HRs and a 95% CI from 13 study names (derived from 12 studies) [30]. Statistical significance was assessed using Z-tests with an alpha level of 0.05.

Heterogeneity was evaluated using the Cochrane Q test and *I*^2^ statistics, with significant heterogeneity defined as *p* < 0.10 or *I*^2^ > 50% [31]. To investigate potential sources of heterogeneity in the relationship between adiponectin levels and mortality, we conducted pre-specified subgroup analyses stratifying by: (a) study site (Asia vs. non-Asia), (b) dialysis modality (HD vs. peritoneal dialysis [PD]), (c) age (<55 vs. ≥55 years), (d) sex proportion (<47% vs. ≥47% female), (e) BMI (<25 vs. ≥25 kg/m^2^), and (f) baseline adiponectin levels (≤20 vs. >20 µg/mL). Clinically relevant cut-off values were established through a comprehensive systematic review of the literature. For each subgroup analysis, we calculated pooled HRs and assessed heterogeneity using *I*^2^ statistics to determine which factors might explain the observed between-study variation.

To assess robustness, we conducted leave-one-out sensitivity analyses and cumulative meta-analyses to evaluate individual study impact and temporal evolution of evidence, respectively. Publication bias was assessed using Begg’s and Egger’s tests through funnel plot analysis, with statistical significance set at *p* < 0.05 (one-tailed).

## 3. Results

### 3.1. Results of Literature Search

**Figure 1:**
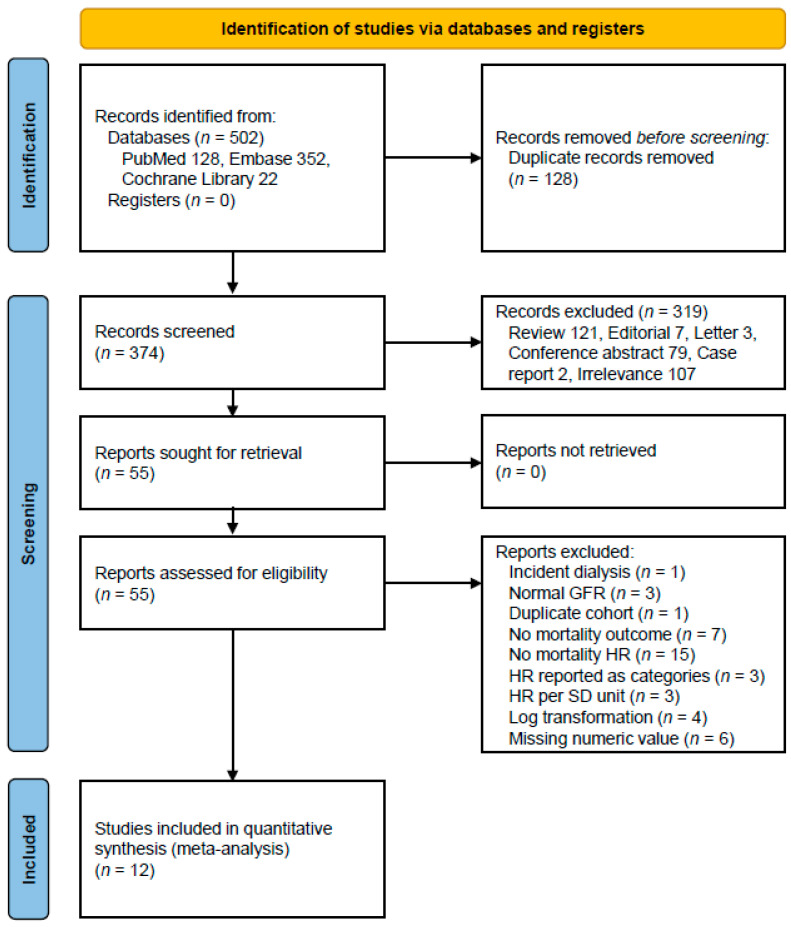
A flow diagram of the study selection. Abbreviations: GFR, glomerular filtration rate; HR, hazard ratio; SD, standard deviation.

Our systematic database search identified 502 potentially relevant articles across multiple databases: PubMed (*n* = 128), Embase (*n* = 352), and Cochrane Library (*n* = 22). After removing 128 duplicate records, we screened 374 unique abstracts. Following the initial screening, 55 articles were retrieved for full-text assessment. Of these, 12 studies met our inclusion criteria [14,15,16,21,28,32,33,34,35,36,37,38], comprising a total study population of 2523 subjects. The detailed study selection process is illustrated in Figure 1.

### 3.2. Characteristics of Included Studies

Our systematic review identified 12 studies, with their key characteristics summarized in Table 1. Six studies [14,16,28,35,36,38] were conducted in Asian countries. Eleven studies included patients with CKD stage 5 or end-stage renal disease on renal replacement therapy [25]: eight [14,21,28,33,34,36,37,38] exclusively on HD, two [16,35] exclusively on PD, and one [15] with both HD and PD patients. Follow-up periods ranged from 1.5 to 10 years, during which all-cause mortality rates varied between 10% and 43%. All studies demonstrated high methodological quality, scoring 8–9 points on the NOS (Supplementary Materials Table S3).

The relationship between adiponectin levels and mortality risk was reported using both unadjusted and adjusted HRs per 1 µg/mL increase in adiponectin (Table 2). The unadjusted HRs revealed conflicting results: three studies (Ohashi, 2008 [14], Markaki, 2012 [15], Rhee, 2015 [21]) found higher adiponectin levels were associated with increased mortality risk, while three others (Park, 2013 [16], Tung, 2015 [35], Zhou, 2016 [36]) reported the opposite. In two studies (Menon, 2006 [32], Abdallah, 2012 [33]), the association between adiponectin levels and all-cause mortality became statistically significant only after adjustment.

### 3.3. Association Between Adiponectin Levels and All-Cause Mortality

**Figure 2:**
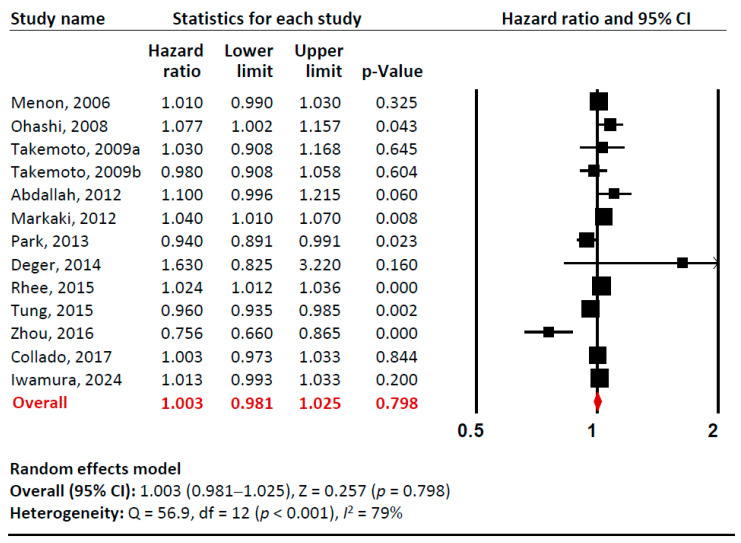
A forest plot of meta-analysis. Adiponectin (per 1 µg/mL increment) and risk of all-cause mortality in patients with chronic kidney disease. Studies included: Menon, 2006 [32]; Ohashi, 2008 [14]; Takemoto, 2009a [28]; Takemoto, 2009b [28]; Abdallah, 2012 [33]; Markaki, 2012 [15]; Park, 2013 [16]; Deger, 2014 [34]; Rhee, 2015 [21]; Tung, 2015 [35]; Zhou, 2016 [36]; Collado, 2017 [37]; Iwamura, 2024 [38]. Abbreviations: CI, confidence interval; df, degrees of freedom.

Our meta-analysis examined the relationship between baseline adiponectin levels (per 1 µg/mL) and all-cause mortality risk in total (Figure 2, Supplementary Materials Figures S1 and S2). Sensitivity analysis confirmed that no single study significantly influenced the overall effect estimate, indicating the robustness of our findings. Cumulative analysis demonstrated gradual stabilization of the pooled effect estimate over time as additional studies were incorporated. Overall, we found no significant association between adiponectin levels and all-cause mortality in CKD patients (pooled unadjusted HR 1.003 [95% CI: 0.981–1.025]). However, there was substantial heterogeneity among studies (*I*^2^= 79%).

**Figure 3:**
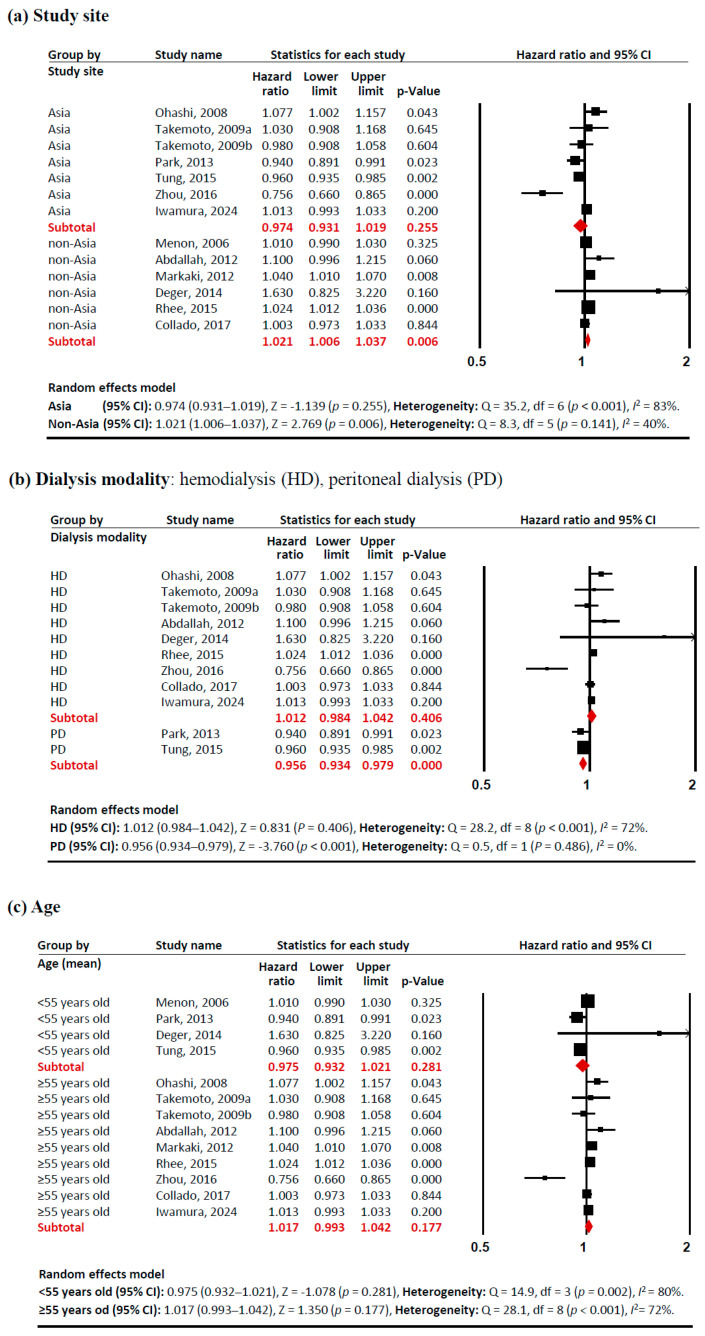

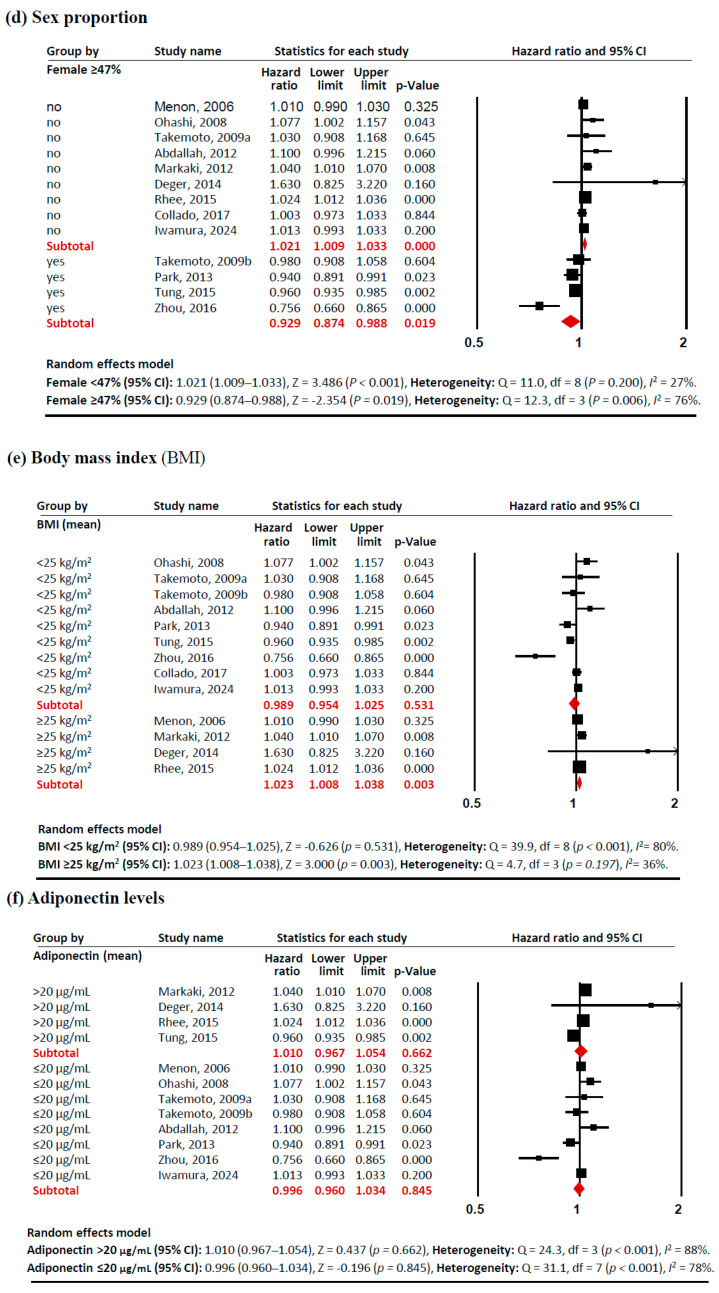
Forest plots of subgroup meta-analysis. Adiponectin (per 1 µg/mL increment) and risk of all-cause mortality in patients with chronic kidney disease. (**a**) study site (Asia, non-Asia), (**b**) dialysis modality (HD, PD), (**c**) age (<55 years old, ≥55 years old), (**d**)sex proportion (<47% vs. ≥47% female), (**e**) BMI (<25 kg/m^2^, ≥25 kg/m^2^), and (**f**) adiponectin levels (>20 µg/mL, ≤20 µg/mL). Studies included: Menon, 2006 [32]; Ohashi, 2008 [14]; Takemoto, 2009a [28]; Takemoto, 2009b [28]; Abdallah, 2012 [33]; Markaki, 2012 [15]; Park, 2013 [16]; Deger, 2014 [34]; Rhee, 2015 [21]; Tung, 2015 [35]; Zhou, 2016 [36]; Collado, 2017 [37]; Iwamura, 2024 [38]. Abbreviations: CI, confidence interval; df, degrees of freedom.

Subgroup analyses revealed several notable patterns that helped explain this heterogeneity (Figure 3). In terms of study sites, studies conducted outside Asia showed a significant positive association between adiponectin levels and mortality risk (HR 1.021 [95% CI: 1.006–1.037]). Similarly, studies with a lower proportion of females (<47%) demonstrated a positive association (HR 1.021 [95% CI: 1.009–1.033]), as did populations with higher BMI (≥25 kg/m^2^) (HR 1.023 [95% CI: 1.023–1.038]). These associations showed consistent effects across studies, with low heterogeneity (*I*^2^ <50%). In contrast, certain subgroups exhibited significant negative associations between adiponectin levels and mortality risk. PD patients showed a protective effect (HR 0.956 [95% CI: 0.934–0.979]) with no heterogeneity (*I*^2^ = 0%). Studies with a higher proportion of females (≥47%) also demonstrated a negative association (HR 0.929 [95% CI: 0.874–0.988]), although with significant heterogeneity (*I*^2^ = 76%). These findings indicate that the relationship between adiponectin and all-cause mortality in CKD patients is significantly modulated by study site, dialysis modality, sex proportion, and BMI, with these factors accounting for much of the observed heterogeneity in the overall analysis.

### 3.4. Publication Bias Analysis

**Figure 4:**
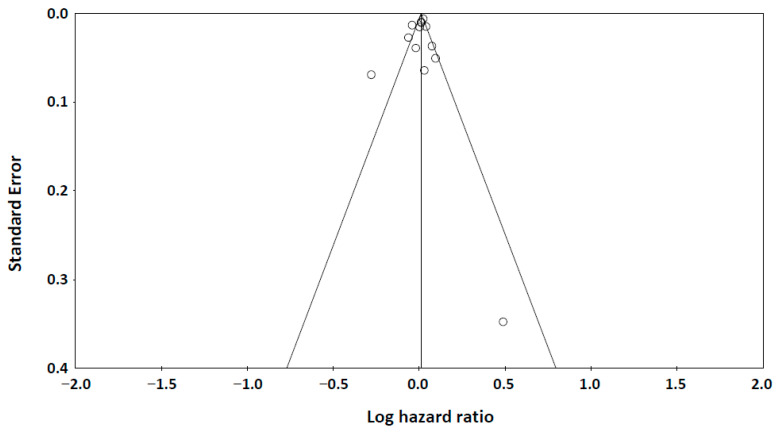
A funnel plot of publication bias. Begg’s test: Kendall’s tau = −0.167, *p* = 0.214 (1-tailed). Egger’s test: intercept = −0.723 (95% Confidence Interval: −2.867, 1.421), *p* = 0.237 (one-tailed).

The funnel plot (Figure 4) showed symmetric distribution of studies examining adiponectin levels and all-cause mortality. Both Begg’s test (*p* = 0.214) and Egger’s test (*p* = 0.237) confirmed no significant publication bias, supporting the robustness of our findings.

## 4. Discussion

This study represents, to our knowledge, the first comprehensive meta-analysis examining the relationship between circulating adiponectin levels and mortality risk in patients with CKD across diverse subgroups. Our analysis revealed that the association between adiponectin levels (per 1 µg/mL increment) and all-cause mortality varies significantly across different patient populations. Specifically, elevated adiponectin levels were associated with increased mortality risk in the non-Asia, higher male proportion, and higher BMI groups. Conversely, higher adiponectin levels correlated with reduced mortality risk in the PD and higher female proportion groups (Figure 3). These findings suggest that elevated adiponectin levels may have a protective effect in specific patient profiles—particularly among young, non-obese, Asian females undergoing PD. However, these interpretations warrant careful consideration, as our analyses were conducted at the aggregate group level rather than the individual patient level, potentially masking important individual variations and interactions.

The elevated adiponectin levels observed in CKD present a complex phenomenon requiring careful interpretation. While Mendelian randomization studies [39,40] suggest adiponectin is unlikely to be a direct causal factor, its elevation may serve as an important biomarker for mortality. Several mechanisms have been proposed to explain this ‘adiponectin paradox’ in CKD patients. Reduced adiponectin clearance corresponding to disease severity appears to be a primary mechanism, while high adiponectin levels may also reflect protein-energy wasting and malnutrition [18] or development of adiponectin resistance [9,41]. Additional proposed mechanisms include broader metabolic dysregulation [42], enhanced production of pro-inflammatory cytokines (particularly IL-6 and TNF-α) [19,43,44], and modification of adiponectin’s protective effects by uremic toxins [19,44]. Our findings suggest an additional perspective regarding the right-arm positioning of the U-shaped mortality risk curve. Our subgroup analysis (Figure 3f) revealed opposing associations with mortality between the low and high adiponectin groups (≤20 µg/mL: HR 0.960; >20 µg/mL: HR 1.010). This pattern suggests a context-dependent relationship: in populations with typically low adiponectin levels (such as healthy individuals), decreased levels are associated with higher mortality risk, while in populations with characteristically high levels (such as CKD patients), further elevation corresponds to increased mortality. This interpretation aligns with previous studies reporting U-shaped associations between adiponectin levels and mortality [41,45,46]. Future research employing non-linear modeling approaches [47] could help identify the optimal adiponectin range and better characterize this U-shaped relationship. Additionally, the ‘adiponectin paradox’ has been observed exclusively in human prospective studies, not in animal models [3], necessitating well-designed clinical trials employing multifactorial analyses or randomized double-blind controls to better understand this species-specific discrepancy.

A key strength of this meta-analysis lies in its comprehensive subgroup analyses. While racial and ethnic data were not available across studies, the geographical subgroup meta-analysis (Figure 3a) unveiled distinct mortality risk patterns: studies from Asian countries demonstrated a trend toward lower mortality risk (HR 0.974), while those from non-Asian countries showed slightly elevated risk (HR 1.021). Although we initially planned to analyze pre-dialysis patients, this was not feasible as only one study examining CKD stages 3–4 was available [32], creating an important knowledge gap regarding adiponectin’s prognostic value in earlier CKD stages. Our findings should be applied cautiously to pre-dialysis populations, as adiponectin-mortality relationships likely differ across the CKD spectrum. The dialysis modality subgroup analysis (Figure 3b) revealed a remarkable contrast: elevated adiponectin levels correlated with increased mortality risk in the HD group (HR 1.012) but decreased risk in the PD group (HR 0.956). This pattern aligns with established demographic differences between dialysis populations, as PD patients generally tend to be younger, more physically independent, and demonstrate better cardiovascular stability compared to HD patients [48,49]. Due to its molecular size (30 kDa), adiponectin is poorly cleared by both HD and PD; therefore, neither modality effectively removes adiponectin from circulation [50]. Elevated adiponectin in both HD and PD patients is primarily due to reduced renal clearance rather than increased production [50,51], with no significant differences having been reported in serum adiponectin levels between HD and PD patients [50,51,52]. Additional subgroup analyses showed protective associations between elevated adiponectin levels and mortality in both younger patients (HR 0.975) (Figure 3c) and non-obese patients (HR 0.989) (Figure 3e).

This study addresses an important topic with significant clinical implications, as understanding the prognostic value of adiponectin levels in CKD could contribute to improved risk stratification for affected patients. Currently, there is no established use of adiponectin levels as a prognostic biomarker in clinical practice for CKD patients. However, our findings suggest that adiponectin levels should be interpreted holistically, considering CKD stage or dialysis modality, concurrent metabolic parameters, and comorbidities, rather than focusing solely on absolute adiponectin values. Given this complexity, a multi-marker approach to risk stratification may be essential for optimal patient care in CKD [16,53].

Several limitations of our systematic review and meta-analysis warrant consideration. First, although publication bias was not significant among included studies, we observed notable omissions of specific numerical data during the exclusion process, particularly regarding non-significant results [17,54,55,56]. Second, the included studies did not adequately represent the complete spectrum of CKD, with particular underrepresentation of early-stage disease, potentially limiting generalizability. Third, we acknowledge that our use of unadjusted HRs in the primary analysis is a limitation, as unadjusted estimates may misrepresent true associations due to the complexity of mortality determinants in CKD patients. However, this methodological decision was necessary due to the substantial heterogeneity in adjustment variables across included studies (Table 2), which would have introduced additional clinical heterogeneity if pooled. We addressed potential confounding through comprehensive subgroup analyses that identified important effect modifiers while maintaining analytical consistency. Additionally, most studies measured adipokine levels at single time points, limiting temporal analysis. Future research should focus on developing non-linear prediction models and multi-factor adjustment models to evaluate multiple adipokine-related biomarkers simultaneously with particular emphasis on including patients across the entire CKD spectrum, especially those in earlier stages.

## 5. Conclusions

This meta-analysis demonstrated that elevated adiponectin levels have varying associations with all-cause mortality across CKD patient subgroups, with protective effects observed in the PD group and the higher female proportion group. These findings suggest that the prognostic value of adiponectin levels in CKD may be significantly modulated by demographic and clinical factors, highlighting the importance of considering patient-specific characteristics when evaluating mortality risk.

## Figures and Tables

**Table 1:** Characteristics of included studies.

Study Name	Site	CKD *	Subjects (n)	Age (Years)	Female (%)	BMI (kg/m^2^)	Adiponectin Method	Adiponectin (µg/mL)	Follow-Up (Years)	Death (n)	NOS Score
Menon, 2006 [32]	USA	Stage 3–4	820	52 ± 12	40.0	27.1	ELISA	12.8 ± 8.0	10	201	9
Ohashi, 2008 [14]	Japan	HD	74	64 ±2	39.2	alive 20.3 ± 0.4, death 20.7 ± 0.8	ELISA	alive 14.2 [9.7–21.3], death 20.5 [14.0–23.5]	3	15	8
Takemoto, 2009 [28]	Japan	HD	68	M 59 ± 14 F 61 ± 8	44.1	M 20.0 ± 2.6 F 18.6 ± 2.4	ELISA	M 9.3 ± 4.3 F 15.7 ± 7.1	8	27	9
Abdallah, 2012 [33]	Egypt	HD	133	55 ± 17	40.6	23.8 ± 3.6	ELISA	18.1 ± 6.8	2	36	9
Markaki, 2012 [15]	Greece	HD	47	63 ± 14	40.4	NR	ELISA	21 ± 12	4.2	18	9
		PD	27	58 ± 16	51.9	NR	ELISA	28 ± 16			
Park, 2013 [16]	Korea	PD	131	51 ± 12	58.0	24.6 ± 3.1	ELISA	alive 19.6 ± 7.4, death 16.7 ± 7.5	5	22	8
Deger, 2014 [34]	USA	HD	98	49 ± 13	33.7	29 [24.2–36.3]	NR	DM−, Ob+ 12 [8.3–23.3] DM−, Ob− 37.7 [23.7–57.4] DM+, Ob+ 40.2 [18.8–61.6] DM+, Ob− 16.6 [9.8–90.3]	6.5	31	8
Rhee, 2015 [21]	USA	HD	501	55 ± 15	43.9	26.4	ELISA	22.6 [13.8–36.3]	1.5	50	8
Tung, 2015 [35]	Taiwan	PD	78	52 ± 13	50.0	23.0 ± 3.4	ELISA	29.5 ± 18.0	3.5	18	9
Zhou, 2016 [36]	China	HD	105	57 ± 14	47.6	21.90 ± 3.98	ELISA	11.1 ± 2.3	5.3	34	9
Collado, 2017 [37]	Spain	HD	220	61 ± 6	30.0	24.3 ± 4.4	RIA	NR	3.2	74	8
Iwamura, 2024 [38]	Japan	HD	221	67 ± 13	37.6	21.8 ± 3.9	HA	20.0 ± 10.8	7	84	9

* CKD stage by KDIGO guideline [25]. Abbreviations: CKD, chronic kidney disease; HD, hemodialysis; PD, peritoneal dialysis; M, males; F, females; BMI, body mass index; NR, not reported; ELISA, enzyme-linked immunosorbent assay; RIA, radioimmunoassay; HA, homogenous assay; DM, diabetes mellitus; Ob, Obese; NOS, The Newcastle–Ottawa Scale.

**Table 2:** Unadjusted and adjusted hazard ratios for all-cause mortality per 1 µg/mL increase in adiponectin.

Study Name	Unadjusted HR (95% CI)	Adjusted HR (95% CI)	Adjustment Variables
Menon, 2006 [32]	1.01 (0.99–1.03)	1.03 (1.01–1.05)	model 1: age, sex, race, BP, protein diet
		1.04 (1.02–1.04)	model 2: model 1+ BMI, systolic BP, CVD, DM, smoking, HDL-C, triglycerides, HbA1_c_, CRP
		1.03 (1.01–1.05)	model 3: model 2+ proteinuria, GFR
Ohashi, 2008 [14]	1.077 (1.002–1.157)	1.1.03 (1.010–1.194)	age, sex
Takemoto, 2009a [28]	M 1.03 (0.91–1.17)		
Takemoto, 2009b [28]	F 0.98 (0.91–1.06)		
Abdallah, 2012 [33]	1.10 (0.996–1.215)	1.030 (1.010–1.050)	Age, hemodialysis duration, CVD, LVH, smoking, CRP
Markaki, 2012 [15]	1.04 (1.10–1.07)	1.08 (1.30–1.12)	model 1: dialysis mode, magnesium, calcium
		1.07 (1.02–1.12)	model 2: model 1+ age, albumin, CRP
Park, 2013 [16]	0.94 (0.89–0.99)	0.94 (0.89–0.99)	Age
		0.93 (0.87–0.99)	Age, albumin
		0.95 (0.89–1.02)	Age, BMI
		0.95 (0.90–1.01)	Age, CRP
Deger, 2014 [34]	1.63 (0.82–3.24)	1.37 (0.66–2.84)	BMI, DM
Rhee, 2015 [21]	1.024 (1.012–1.036)	1.023 (1.011–1.035)	model 1: age, sex, race, ethnicity, dialysis vintage
		1.022 (1.010–1.035)	model 2: model 1+ DM, albumin, TIBC, creatinine, WBC, phosphate, hemoglobin, nPCR
Tung, 2015 [35]	0.96 (0.94–1.00)		
Zhou, 2016 [36]	0.756 (0.660–0.865)	0.832 (0.696–0.995)	age, diastolic BP, ABI, albumin, CRP
Collado, 2017 [37]	1.003 (0.973–1.033)		
Iwamura, 2024 [38]	1.013 (0.993–1.033)		

Abbreviations: HR, hazard ratio; CI, confidence interval; M, males; F, females; BP, blood pressure; BMI, body mass index; CVD, cardiovascular diseases; DM, diabetes mellitus; HDL-C, high-density lipoprotein cholesterol; HbA1c, glycated hemoglobin; CRP, C-reactive protein; GFR, glomerular filtration rate; LVH, left ventricular hypertrophy; TIBC, total iron-binding capacity; WBC, white blood cell count; nPCR, normalized protein catabolic rate; ABI, ankle-brachial index.

## Data Availability

All data described in this study are available within this article or in the Supplementary Materials.

## Supplementary Materials

The following supporting information can be downloaded at: https://www.mdpi.com/article/10.3390/metabo15040230/s1, Table S1: PRISMA 2020 Checklist; Table S2: PRISMA 2020 for Abstracts Checklist; Table S3: Newcastle-Ottawa Quality Assessment Scale for Cohort Studies; Figure S1: Sensitivity Analysis; Figure S2: Cumulative Analysis.

## Abbreviations

The following abbreviations are used in this manuscript:

CKD	Chronic Kidney Disease
HD	Hemodialysis
PD	Peritoneal Dialysis
BMI	Body Mass Index
HR	Hazard Ratio
CI	Confidence Interval
PRISMA	Preferred Reporting Items for Systematic Reviews and Meta-Analyses
MOOSE	Meta-analysis of Observational Studies in Epidemiology
KDIGO	Kidney Disease Improving Global Outcomes
NOS	The Newcastle–Ottawa Scale
