# Supplementary material for: Adiponectin and All-Cause Mortality in Patients with Chronic Kidney Disease: A Systematic Review and Meta-Analysis
Source: Metabolites. 2025 Mar 27;15(4):230. doi: 10.3390/metabo15040230 (PMC12028947; doi:10.3390/metabo15040230)
Supplement: Supplementary file 1 [file metabolites-15-00230-s001.zip › Table S3_R1.pdf]

Table S3. Newcastle-Ottawa Quality Assessment Scale for Cohort Studies

	Selection				Comparability	Outcome			Total
Studies	Representative of the exposed cohort	Selection of the nonexposed cohort	Ascertainment of exposure	Outcome of interest was not present at the start of the study	Control of important and additional factors	Assessment of outcome	Follow-up enough for outcomes to occur	Adequacy of follow-up of cohorts	
Menon, 2006 [32]	★	★	★	★	★★	★	★	★	9
Ohashi, 2008 [14]	★	★	★	★	★	★	★	★	8
Takemoto, 2009 [28]	★	★	★	★	★★	★	★	★	9
Abdallah, 2012 [33]	★	★	★	★	★★	★	★	★	9
Markaki, 2012 [15]	★	★	★	★	★★	★	★	★	9
Park, 2013 [16]	★		★	★	★★	★	★	★	8
Deger, 2014 [34]	★	★	★	★	★	★	★	★	8
Rhee, 2015 [21]	★	★	★	★	★★	★		★	8
Tung, 2015 [35]	★	★	★	★	★★	★	★	★	9
Zhou, 2016 [36]	★	★	★	★	★★	★	★	★	9
Collado, 2017 [37]	★	★	★	★	★	★	★	★	8
Iwamura, 2024 [38]	★	★	★	★	★★	★	★	★	9
