# Supplementary figures and images for: Adiponectin and All-Cause Mortality in Patients with Chronic Kidney Disease: A Systematic Review and Meta-Analysis
Source: Metabolites. 2025 Mar 27;15(4):230. doi: 10.3390/metabo15040230 (PMC12028947; doi:10.3390/metabo15040230)

**Figure S1.** Sensitivity Analysis

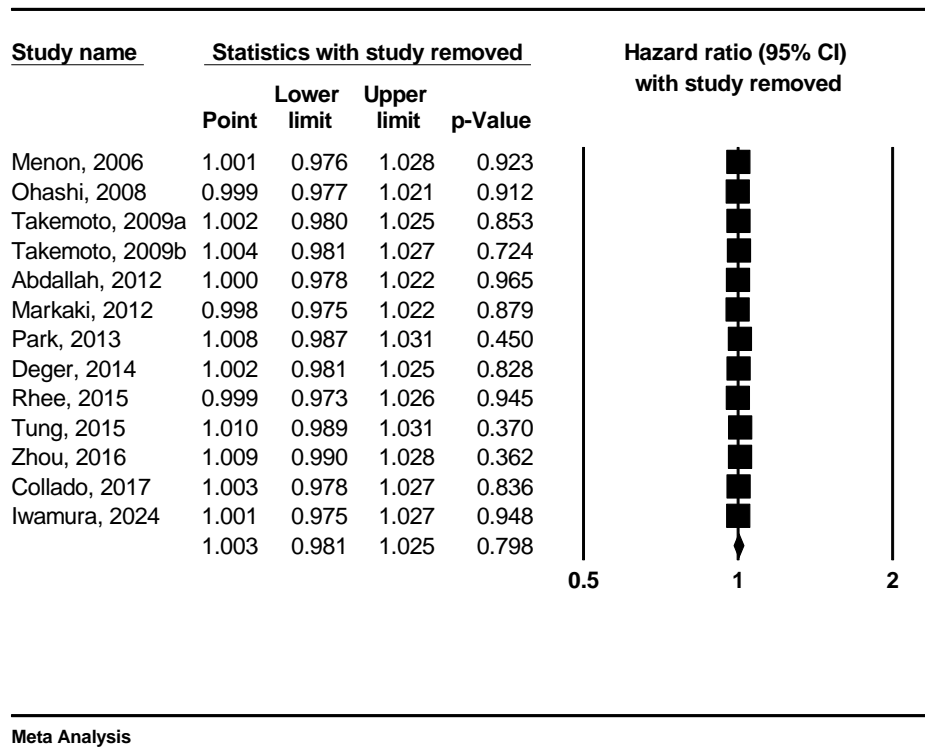

Supplement: Supplementary file 1 [file metabolites-15-00230-s001.zip › Figure S1.pdf]

**Figure S2. Cumulative Analysis**

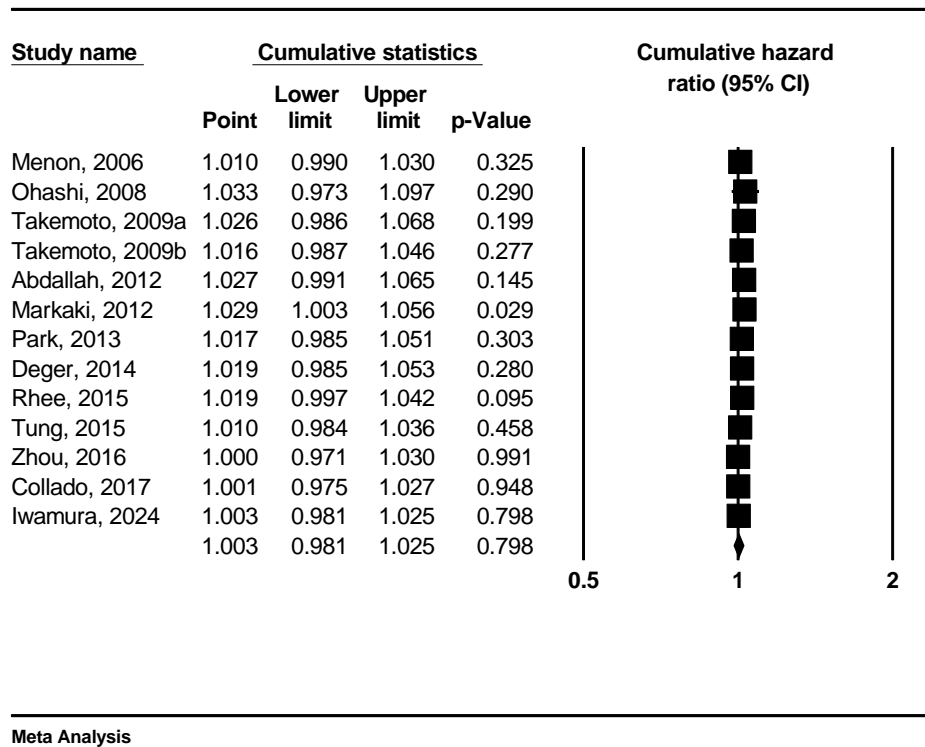

Supplement: Supplementary file 1 [file metabolites-15-00230-s001.zip › Figure S2.pdf]
